# Enhancing tertiary cardiology triage with vectorcardiographic features: a machine learning approach using real-world data

**DOI:** 10.1016/j.clinsp.2025.100856

**Published:** 2026-01-08

**Authors:** Lucas José da Costa, Vinicius Ruiz Uemoto, Mariana FN de Marchi, Renato de Aguiar Hortegal, Renata Valeri de Freitas

**Affiliations:** a Instituto Internacional de Neurociência Edmond e Lily Safra, Macaíba, RN, Brazil; b Instituto Dante Pazzanese de Cardiologia, São Paulo, SP, Brazil; c Escola Politécnica da Universidade de São Paulo, São Paulo, SP, Brazil

**Keywords:** Cardiology, Survival prediction, Machine learning, Vectorcardiogram, Electrocardiogram, Global electric heterogeneity, Tertiary care

## Abstract

**Objective:**

To assess whether electrocardiographic markers of Global Electrical Heterogeneity (GEH) improve the identification of patients requiring tertiary care, either alone or combined with an explainable machine learning model, compared with standard ECG features and clinical risk factors in a real-world tertiary cardiology population.

**Methods:**

Patients were forwarded to a specific evaluation in a cardiology-specialized hospital performed an ECG and data collection. A series of follow-up attendances occurred in periods of 6-months, 12-months and 15-months to check for cardiovascular-related events (mortality or new nonfatal cardiovascular events (Stroke, MI, PCI, CS), as identified during 1-year phone follow-ups. The first attendance ECG was measured by a specialist and processed in order to obtain the Global Electric Heterogeneity (GEH) using the Kors Matriz. The ECG measurements, GEH parameters, and risk factors were combined for training multiple instances of XGBoost decision tree models. Each instance was optimized for the AUCPR, and the instance with the highest AUC was chosen as representative of the model. The importance of each parameter for the winner tree model was compared to better understand the improvement from using GEH parameters.

**Results:**

GEH parameters were statistically significant in this population (*p* < 0.001), particularly the QRST angle and SVG magnitude. The combined model integrating GEH, standard ECG features, and clinical risk factors achieved the best performance, with a sensitivity of 94.1 %, specificity of 30.8 %, AUC of 67.6 %, and F2 score of 0.62. SVG feature importance and SHAP analyses were consistent with the statistical findings, indicating that the model's decision patterns align with clinically relevant information and reinforce the role of GEH features. The modeling approach was carefully designed to prevent overfitting, ensure generalizability, and facilitate implementation through its decision tree architecture.

**Conclusion:**

VCG-derived features may improve the identification of patients requiring tertiary care, either alone or integrated into an explainable and robust machine learning model trained on real-world data. Its clinical value will ultimately depend on prospective validation and seamless integration within existing care pathways.

## Highlights

• ML model uses VCG-derived GEH to predict tertiary care (AUC 68 %, Sens 94 %) for triage.
• Interpretable XGBoost prioritizes GEH and prior PCI, matching clinical risk factors.
• High sensitivity and low specificity highlight its use as a screening support tool.
• SHAP confirms robustness of SVG, QTc, and age across several sensitivity scenarios.
• VCG biomarker extraction enables low-cost, scalable support for clinical triage use.

## Introduction

Cardiovascular diseases are the main cause of death around the world.^[^^1^ In 2022, 28 % of the deaths in Brazil had cardiovascular causes.^[^^2^ Being able to detect and predict cardiovascular events, since one-third-of the deaths due to coronary disease and strokes occur in people under 70-years,^[^^3^ is extremely positive and lifesaving, particularly when it is done using non-invasive exams.

The past decade saw significant technological advances, especially in computation and machine learning, with some of these having similar or better accuracy than specialists when diagnosing a disease. However, a lot still needs to be done in order to improve the quality of the models and their validation in clinical practice.^[^^4^

Although the GRACE and TIMI scores are well- established tools for prognostic risk stratification in patients with Acute Coronary Syndromes (ACS), as endorsed by both the European Society of Cardiology (ESC)^[^^5^ and the American College of Cardiology/American Heart Association (ACC/AHA) guidelines,^[^^6^ their real-world applicability is often limited outside of specialized care environments. These scores require the integration of multiple clinical and laboratory parameters, including troponin levels, serum creatinine, Killip class, and detailed ECG interpretation, which are not always available at the point of first contact. In particular, non-specialized settings such as primary care facilities, ambulance units, and telemedicine platforms often lack the resources or personnel necessary to obtain and interpret these data in real time. In those environments, models that automatically extract ECG and VCG features may provide a complementary strategy to support early risk stratification. While such models do not replace established clinical scores, they may help bridge the gap in initial triage when laboratory results or full clinical assessments are delayed or inaccessible.

Previous studies have emphasized the predictive importance of ECG-derived features in cardiovascular risk assessment.^[^^7^^,^^8^ Notably, the incorporation of Global Electrical Heterogeneity (GEH) metrics such as spatial QRS-T angles and SVG magnitude has shown promise in enhancing disease detection when combined with clinical data.^[^9, 10, 11 Additionally, the reproducibility and consistency of GEH measurements across diverse populations have been demonstrated in large cohort studies, such as the Multi-Ethnic Study of Atherosclerosis, reinforcing their potential utility in clinical practice.^[^^12^

In support of triage innovation, recent work has demonstrated that artificial intelligence-enabled ECGs can effectively screen for cardiac contractile dysfunction, offering scalable, low-cost tools for identifying patients at risk of heart failure using standard ECG signals.^[^^13^

Building on this evidence, the present study explores the potential of GEH markers in a novel triage context, assessing whether electrocardiographic markers of Global Electrical Heterogeneity (GEH) improve the identification of patients requiring tertiary care either alone or in combination with an explainable machine learning model compared with standard ECG features and clinical risk factors in a real-world tertiary cardiology population.

## Materials and methods

### Population

This study was performed in a population attended from May to August 2017 in a first-attendance ambulatory in Dante Pazzanese Institute of Cardiology (IDPC), a tertiary/quaternary public healthcare center in São Paulo. Local institutional and national review boards approved the data collection protocols (CAAE 76085317.5.000.5462). The ambulatory in question focuses on triaging patients referred from primary and secondary care centers.

All patients who attended the ambulatory during the specified time period were contacted through phone calls during the follow-ups, and all patients who did not respond to any follow-ups (and therefore the outcome was unavailable) were excluded from the study's population. 303 subjects underwent a first attendance; after the 6-months follow-up, 293-subjects' outcomes were confirmed by phone call. For the 1-year follow-up 274-subjects' outcomes were confirmed by phone call.

To assess the potential impact of missing outcome data on model performance and generalizability, the authors conducted a sensitivity analysis using best-case and worst-case imputation scenarios. In the best-case scenario, all patients lost to follow-up were assumed not to have experienced the outcome of interest; in the worst-case scenario, they were assumed to have experienced it. Predictive models were retrained under both conditions, and performance metrics ‒ including classification accuracy, Area Under the ROC Curve (AUC), and variable importance ‒ were recalculated. This analysis was performed to evaluate the robustness of the model under extreme assumptions regarding missing outcomes and to transparently address potential bias introduced by incomplete follow-up.

### Data collection

Only patients with complete data were included in this study. Since the required information was already integrated into the hospital's standard procedures, no additional data collection was necessary. All data from each patient was collected from 2 main sources: a 12-lead ECG exam and the patient's previous cardiovascular events' history. The electrocardiography signals were obtained in 7 seconds acquisition of a 12-lead exam at a sampling rate of 240 Hz, the traces were stored so they could be properly pre-processed, and every exam was analyzed by a cardiology specialist who was also responsible for measuring the standard parameters from the ECG, as well as marking the baseline and peaks necessary for GEH computation. Patient backgrounds were obtained during anamnesis, and all data were saved in table format.

### Data pre-processing

The raw ECG traces needed to be pre-processed so that they could be used to estimate the patient's vectorcardiogram through the Kors' transformation method.^[^^14^ The VCG would then be used to calculate the Global Electric Heterogeneity markers as was done by Waks et al.^[^^9^

**Fig. 1:**
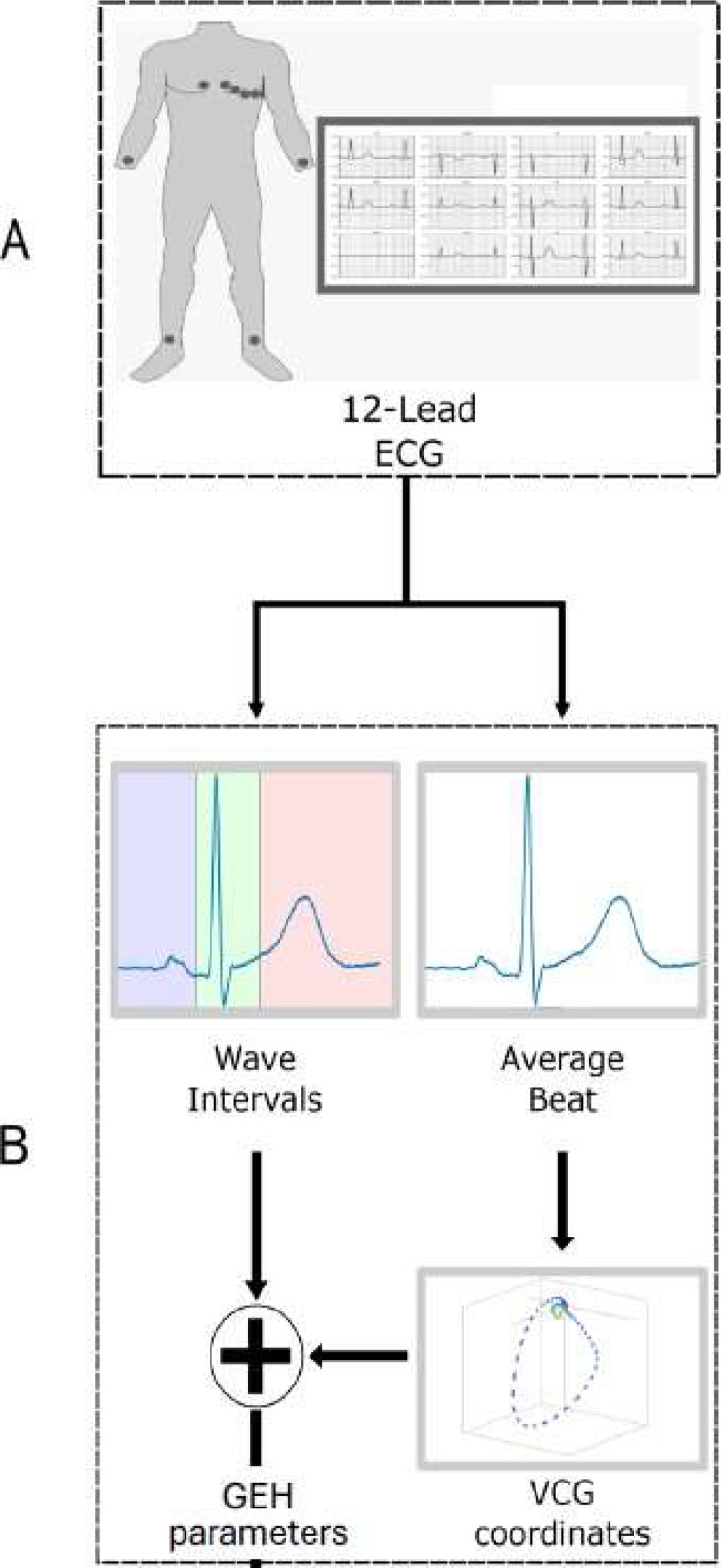
Process for obtaining the GEH parameters. 12-lead ECG (A), measurement of the wave intervals and average beat computation. Kors matrix is applied to generate the Frank leads and Vectocardiogram (B), Computation of the vectocardiographic features and Spatial Vector Gradient (C).

**Fig. 2:**
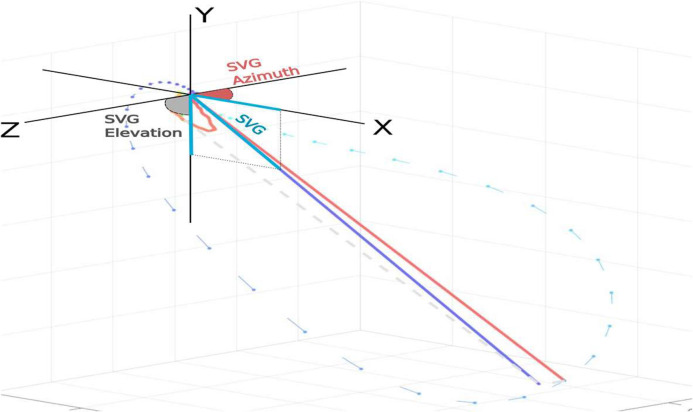
Representation of SVG Elevation and Azimuth angles.

The ECG wave intervals were measured by a cardiology specialist using an in-house developed ECG specialized software that marked the timestamp of the baseline, beginning, peak and ending of each wave of, at least, 3-cardiac cycles. Then, the median timestamps were submitted to an adapted version of Tereshchenko's GEH Analysis algorithm^[^^15^ that converts the ECG to VCG and then extracts the GEH parameters (Fig. 1, Fig. 2).

### Parameter sets

To investigate the effect of each type of parameter, the authors trained 4 machine learning models with different parameter sets. Three of those models contained only one category of features: Risk factors (R), Standard Electrocardiographic measurements (S), or Global heterogeneity measurements (G). The first three models serve as a performance reference to how well a model with only those parameters set could perform, while the fourth model (SRG) is a combination of all the categories to evaluate if knowing the GEH parameters could lead to better predictions.

### XGBoost

The choice to use a decision tree with XGBoost as a machine learning model is due to its simple architecture and ease of interpretation of results, while having a great performance, especially when working with tabular data.^[^^16^^,^^17^ The data were randomly divided in 70 % for training and 30 % for testing. Due to the training data being unbalanced by a ratio of 5 negative outcomes to 1 positive, they needed to pass by both bootstrap oversampling and undersampling before the learning process in order to avoid biases. The authors applied this hybrid strategy using the ovun.sample() function from the ROSE package in R (method = “both”). This approach balances the outcome classes by randomly duplicating minority class observations and removing a subset of majority class cases, without generating synthetic data.

**Fig. 3:**
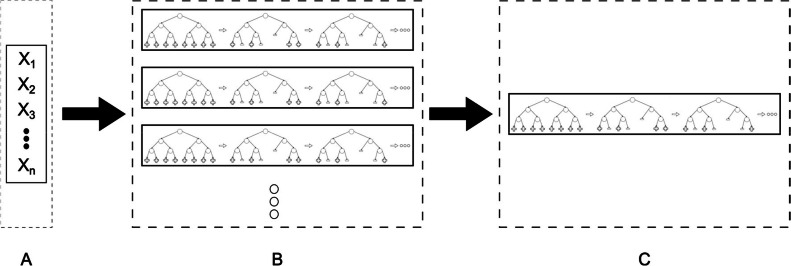
Overall process to obtain the model representative. (A) The parameter set that will be used for training (B) 50 different instances of XGBoost trees were trained to mitigate the randomness influence. Each instance using the AUCPR as metric (C) The instance with the highest AUC is chosen as representative of the model.

The choice of this combined approach was based on its ability to mitigate potential bias introduced by heavily skewed class distributions, which is especially important when training classifiers such as XGBoost that can otherwise be biased toward the majority class. By balancing the classes, the model is better able to learn patterns from both classes, improving generalizability and ensuring more reliable evaluation metrics such as AUCPR. To achieve optimal performance in the quality analysis,^[^^18^ each XGBoost model underwent κ-fold cross-validation, with κ = 5-folds. For each fold, the data was randomly split without stratification into training and validation subsets, ensuring that the learning rate and the number of boosting rounds were tuned based on the minimal difference between the mean and standard deviation of the test's AUCPR. This approach allowed us to identify the best-performing learning configuration while minimizing overfitting. The final model was then retrained using the entire training dataset and evaluated on the test set, with SHAP values computed to interpret feature contributions. To avoid randomness influencing model performance, 50 instances of the XGBoost tree were created per model, as shown in Fig. 3. All instances were trained with the optimal learning rate, number of rounds, and using AUCPR as the evaluation metric since detecting positive outcomes is a higher priority than negative outcomes for a triage application.. ^[^^19^ From all the 50 instances in one model, the one with the highest AUC was selected to be the final XGBoost tree for the model. With this selection method is possible to have a model that prioritizes the positive outcomes, but without ignoring the prediction of negative outcomes.

### Model’s performance

Traditionally, the way to choose the best model is by using the Area Under the Receiver Operator Characteristic (AUROC) as a criterion. However, in view of the fact it is easier for the clinician to interpret a binary output, the authors used the same principle as in the study published by Pollard et al.^[^^20^ which aimed for the maximum sensitivity threshold from the AUROC, since the goal was to screen for all the positive cases. Since that study had a bigger population, this didn't necessarily implied on a high false positive rate. In the present study, due to a relatively small number of outcomes, in order to have a practical value of sensitivity, the choice of 90 % instead of 100 % was necessary so the threshold couldn't be low enough to consider all predictions as positive, overloading the healthcare system. The threshold can be chosen as a bare minimum or as a maximum for the prediction be considered as positive, depending on which case will lead to a higher AUC. The F2 score was used to compare the models since it is a suitable and effective metric for unbalanced data, especially when the goal is to maximize recall, such as in medical triage applications.

### Feature importance and explainability analysis

To identify which clinical and electrophysiological features most strongly influenced model predictions, the authors quantified feature relevance using two complementary methods: gain-based importance from the XGBoost algorithm and SHAP (Shapley Additive exPlanations) analysis.

First, the authors computed gain-based importance, which is derived from the decision-tree architecture of the XGBoost model. In this context, gain reflects the average improvement in model performance (measured by a reduction in classification error) when a specific variable is used to split the data within the trees. In practical terms, a variable with higher gain is one that consistently improves the model's ability to discriminate between patients with and without the clinical outcome, across multiple branches of the decision tree ensemble. While widely used in predictive modeling, gain-based importance may be biased toward features with higher variability or more distinct categories.

To enhance the transparency of the model and mitigate potential biases, the authors supplemented this analysis with SHAP values, which offer a more interpretable and individualized assessment of variable impact. Unlike gain, which is aggregated at the model level, SHAP allows both global interpretability (which variables contribute most across all patients) and local interpretability (why a specific patient received a given prediction).

## Results

Table 1 presents the demographic characteristics, risk factors, and ECG and VCG parameters of the study cohort, stratified by the outcome groups (negative and positive). The study population comprised 274 individuals, with a median age of 59-years (interquartile interval: 51.0 to 67.0) and a body mass index of 26.9 (IQR: 24.2 to 30.4). Of these individuals, 52.2 % were female. No significant differences in sex distribution or BMI were observed between the negative and positive outcome groups (*p* = 0.112 and *p* = 0.290, respectively). In terms of comorbidities, 69.0 % of the individuals presented with hypertension, emphasizing the high prevalence of this condition within the study population. Moreover, 27.7 % of the participants had diabetes. Within this cohort, it is noteworthy that a significant proportion had experienced previous cardiac events. Specifically, 28.1 % of the participants had a documented history of Previous Myocardial Infarction (MI), indicating a considerable burden of ischemic heart disease. Additionally, 14.6 % had undergone previous Percutaneous Coronary Intervention (PCI), highlighting the need for coronary revascularization procedures. Furthermore, 11.3 % had a history of Previous Cardiac Surgery, reflecting the prevalence of complex cardiovascular conditions needing surgical interventions. The prevalence of previous MI was significantly higher in the positive outcome group (51.0 %) compared to the negative outcome group (22.9 %), with a p-value of <0.001. Similarly, previous PCI was more common in the positive group (39.2 %) compared to the negative group (9.0 %) with a p-value of < 0.001.

**Table 1:** Values are n ( %) for categorical variables, median [IQR] for continuous non-normal variables, and mean (SD) for continuous normal variables.

	Outcome			
	Total	Negative	Positive	p-value
**N**	274	223	51	
**Sex = *F***	143 (52.2)	122 (54.7)	21 (41.2)	0.112
**Age, y**	59.0 [51.0, 67.0]	58.0 [48.5, 66.0]	63.0 [57.5, 69.5]	0.001
**BMI, kg/m**	26.9 [24.2, 30.4]	26.5 [24.1, 30.5]	27.5 [25.0, 30.4]	0.290
**Risk factors**				
Previous cardiac surgery	31 (11.3)	19 (8.5)	12 (23.5)	0.005
Previous MI	77 (28.1)	51 (22.9)	26 (51.0)	<0.001
Previous PCI	40 (14.6)	20 (9.0)	20 (39.2)	<0.001
Previous stroke	18 (6.6)	13 (5.8)	5 (9.8)	0.471
Hypertension	189 (69.0)	151 (67.7)	38 (74.5)	0.436
Diabetes	76 (27.7)	51 (22.9)	25 (49.0)	<0.001
**Standard ECG parameters**				
P-wave interval, ms	102.2 [96.0, 112.0]	102.0 [95.3, 111.2]	104.0 [97.0, 113.7]	0.364
PR segment interval, ms	163.0 [146.8, 181.7]	163.0 [146.9, 182.3]	165.0 [145.7, 180.7]	0.757
QRS interval, ms	92.0 [83.0, 100.6]	91.3 [82.7, 100.2]	92.3 [83.8, 104.7]	0.283
QT interval, ms	386.0 [364.6, 410.1]	385.3 [364.8, 407.5]	387.3 [361.8, 421.0]	0.741
Corrected QTi, ms	408.7 (33.7)	407.7 (34.1)	413.0 (31.8)	0.312
RR interval, ms	920.8 [793.7, 1047.9]	915.3 [805.2, 1035.4]	933.3 [745.8, 1075.0]	0.744
**GEH Parameters**				
Peak QRST angle	44.8 [25.7, 90.2]	37.9 [25.6, 81.1]	69.9 [35.4, 120.4]	0.001
Area QRST angle	66.1 [41.2, 111.0]	60.4 [38.8, 103.3]	92.2 [52.7, 137.5]	0.007
Peak SVG Azimuth	4.4 [−6.4, 21.3]	4.7 [−6.5, 19.6]	4.4 [−4.2, 34.1]	0.347
Area SVG Azimuth	−11.6 [−22.8, 3.8]	−9.7 [−20.8, 4.3]	−16.1 [−24.7, −2.1]	0.179
Peak SVG Elevation	69.7 [60.9, 77.5]	68.6 [60.2, 76.8]	71.9 [64.0, 81.3]	0.046
Area SVG Elevation	63.9 [54.0, 77.8]	63.5 [55.0, 77.7]	67.6 [47.8, 78.7]	0.864
Peak SVG, mV	1.7 [1.3, 2.1]	1.7 [1.3, 2.1]	1.5 [1.3, 1.8]	0.107
VmQTI, mVms	98.3 [78.9, 118.2]	100.1 [77.6, 118.8]	89.6 [80.9, 111.7]	0.551
SVG, mV*ms	61.1 [40.3, 83.7]	65.0 [42.4, 85.9]	45.6 [29.5, 61.9]	0.001

BMI indicates Body Mass Index; MI, Myocardial Infarction; PCI, Percutaneous Coronary Intervention; SVG, Spatial Ventricular Gradient; VmQTI, Vector Magnitude QT Integral.

These findings emphasize the importance of targeted triage and comprehensive management strategies for individuals with cardiovascular diseases, particularly considering the substantial prevalence of risk factors such as hypertension and diabetes, as well as the notable occurrence of prior cardiac events in this population. Previous cardiac surgery and diabetes also had a higher prevalence in the positive outcome group, with 23.5 % versus 8.5 % for the Previous CS and 49.0 % versus 22.9 % for diabetes with p-values of 0.005 and < 0.001, respectively. No significant differences were found for previous stroke or hypertension between the two groups. The ECG parameters, including P-wave interval, PR segment interval, QRS interval, QT interval, corrected QTi, and RR interval, showed no significant differences between the negative and positive outcome groups. For instance, the QRS interval was 92.0 ms (IQR: 83.0, 100.6) in the total cohort, with no significant difference between groups (*p* = 0.283).

Among the Global Electrical Heterogeneity (GEH) parameters, significant differences were observed in several measures. The peak QRS-T angle was significantly higher in the positive outcome group (69.9 [IQR: 35.4, 120.4]) compared to the negative group (37.9 [IQR: 25.6, 81.1]) with a p-value of 0.001. The area QRS-T angle also showed a significant difference, being higher in the positive group (92.2 [IQR: 52.7, 137.5]) compared to the negative group (60.4 [IQR: 38.8, 103.3]) with a p-value of 0.007. The peak SVG elevation was significantly greater in the positive group compared to the negative group, with p-values of 0.046. Additionally, the SVGs were significantly different between the groups, 65.0 [IQR: 42.4, 85.9] vs. 45.6 [IQR: 29.5, 61.9] for the positive group, with p-values of 0.001. This SVG value for the population without events is consistent with the ones published in^[^^21^ for normal beats of the participants in the Multi-Ethnic Study of Atherosclerosis.

Fig. 5 illustrates the global feature importance as determined by the SHAP (SHapley Additive ex-Planations) values across the study cohort. This visualization offers a comprehensive view of how each clinical, electrocardiographic (ECG), and vectorcardiographic (VCG) parameter contributed to the predictions of the XGBoost model. Among the most influential features were Wilson SVG magnitude (*μ*Vms), age, QTc interval, previous Percutaneous Coronary Intervention (PCI), and the peak QRS-T angle. These parameters demonstrated the highest mean absolute SHAP values, indicating their significant roles in model decision-making.

Notably, several GEH parameters, such as the peak and area QRS-T angle, SVG magnitude, and azimuth and elevation components, were ranked among the top contributors, reinforcing their clinical relevance in characterizing ventricular electrical heterogeneity. Prior cardiac events, including previous Myocardial Infarction (MI) and PCI, also showed a strong influence, aligning with the statistical findings presented in Table 1.

To evaluate the robustness of the predictive model in the presence of missing outcome data, the authors conducted a sensitivity analysis using three scenarios: complete-case analysis (*n* = 274), best-case imputation (missing outcomes assumed negative), and worst-case imputation (assumed positive). Across all scenarios, the SRG model consistently outperformed all other models in accuracy, specificity, and F1 score, while maintaining high sensitivity (> 91 %). Notably, the SRG model achieved the highest accuracy of 65.9 % and specificity of 60.0 % under best-case conditions, and remained superior even in the worst-case scenario (accuracy: 54.9 %, specificity: 41.8 %). To assess the stability and clinical transparency of the model, the authors also performed SHAP (SHapley Additive exPlanations) analysis under each scenario. The most impactful predictors, Wilson's spatial ventricular gradient, age, QTc interval, and QRST angle were consistently ranked highest, underscoring the robustness of the model's internal logic. These findings support the complementary nature of combining standard ECG parameters, clinical risk factors, and global electrical heterogeneity metrics into a unified, interpretable model that remains stable even when assumptions about missing data are varied.

**Fig. 4:**
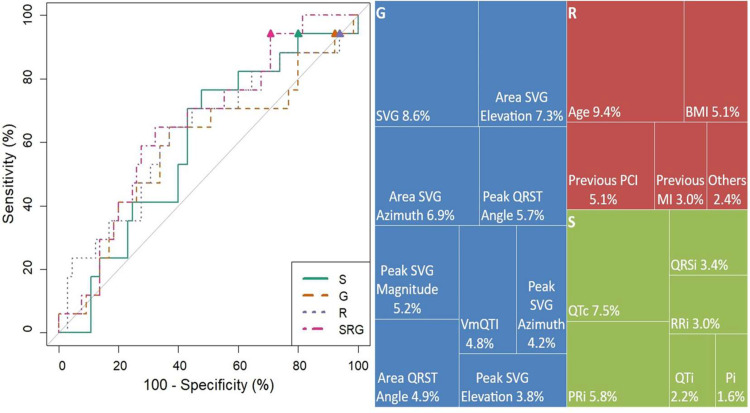
Comparison of the ROC of the models and importance of the features for the winner model. (a) Final AUC of each model as described in 2. S, Standard ECG model; R, Risk factors model; G, Global heterogeneity model; SRG, Combined model with all the S, R and G model features. (b) Importance (gain) for all parameters used to build the SRG model.

In Fig. 4a and Table 2 it is shown the ROCs and the performance metrics of four different machine learning models used for triage are shown, including F2 Score, AUC, sensitivity, and specificity. Model SRG achieved the highest F2 score of 0.62, indicating a better balance between precision and recall compared to the other models. Model SRG also had the highest AUC at 67.6 %, suggesting superior overall performance in distinguishing between positive and negative cases. All models maintained the same sensitivity of 94.12 %, indicating a high true positive rate across the board. Model SRG again outperformed the other models with a specificity of 30.77 %, which is significantly higher than Model S (20.00 %), Model R (6.15 %), and Model G (3.08 %). Overall, Model SRG showed the best performance for triage, with the highest F2 score and AUC, and the best balance between sensitivity and specificity among the models evaluated. Fig. 4b shows that the results indicate that the winning model (Model SRG) relies heavily on Global Heterogeneity features, with the highest gain importance being attributed to SVG (8.6 %), Area SVG Elevation (7.3 %), and Area SVG Azimuth (6.9 %). Demographics and Risk features, particularly Age (9.4 %) and Previous PCI (5.1 %), also have a significant impact on the model. Standard ECG features contribute less to the overall model's performance, even though some intervals, like with QTc (7.5 %) and PRi (5.8 %) were the third and sixth most important values.

**Fig. 5:**
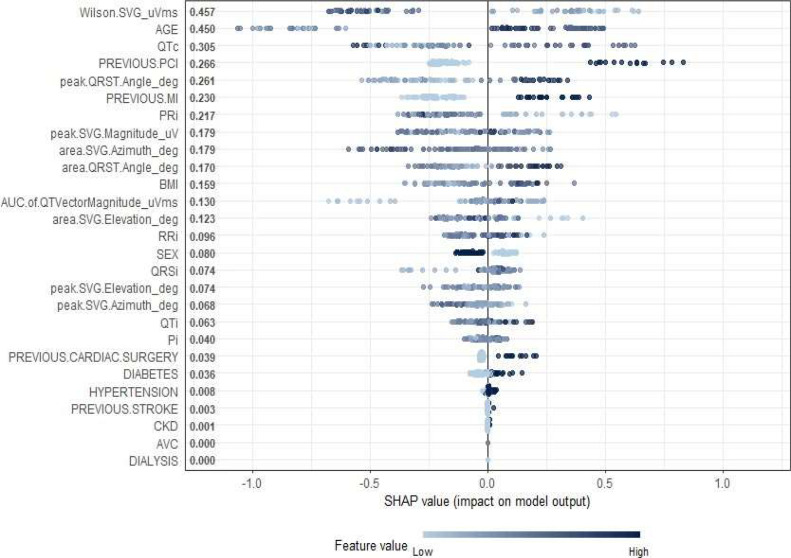
SHAP summary plot highlighting the most influential predictors in the model. Wilson SVG magnitude, age, and QTc interval had the highest mean absolute SHAP values, indicating they contributed most to the model’s output. The color gradient represents the original feature values (low in light blue, high in dark blue), and each point corresponds to an individual prediction.

**Table 2:** Scores for each one of the built models. Each model used one or more sets of the following parameters: Standard ECG parameters (S), GEH calculated (G), and Risk Factors (R).

Model	F2 Score	AUC ( %)	Sensitivity ( %)	Specificity ( %)
S	0.59	59.5	94.12	20.00
G	0.54	54.0	94.12	3.08
R	0.55	62.5	94.12	6.15
SRG	0.62	67.6	94.12	30.77

## Discussion

Cardiovascular disease remains a significant and costly public health challenge. Within high-demand healthcare systems, determining the appropriate level of care for each patient is complex, particularly in referral settings where most patients present with known or suspected cardiovascular conditions and abnormal ECG findings. In this context, computational decision-support methods offer valuable assistance by identifying patients who truly require advanced evaluation. The integration of additional parameters derived from standard ECGs, such as Global Electrical Heterogeneity (GEH) metrics, may enhance triage decisions in a cost-effective manner.

The present analysis identified significant differences in several risk factors and GEH parameters between patients with and without adverse outcomes, highlighting the predictive potential of these measures. Notably, Vectorcardiographic (VCG) features post-processed from standard ECGs outperformed traditional ECG parameters in stratifying patients within the referred population. However, the strong influence of features such as age raises ethical concerns about potential bias ‒ particularly the risk of over-referral in older patients. High SHAP values, XGBoost feature importance linked to age may reflect clinical reality but also underscore the need for fairness-aware model development and validation.

In terms of model performance, while the baseline model using only standard clinical features achieved modest results, the inclusion of GEH parameters improved performance considerably. The combined model achieved an AUC=67.6 %, an F2 score of 0.62, and a specificity of 30.77 %, while maintaining a high sensitivity of 94.12 %. This trade-off favors minimizing false negatives, a clinically critical choice in triage, where failing to detect severe cases could have life-threatening consequences. However, the authors acknowledge that high sensitivity comes at the cost of lower specificity, which increases the risk of over-referral. This limitation is particularly relevant in resource-constrained public healthcare systems and must be addressed in future validation cohorts.

The use of a decision tree model in this study provided interpretable insights into which GEH features contributed most to triage decisions. Key predictive features included the peak magnitude of the Spatial Ventricular Gradient (SVG), overall SVG magnitude, and SVG azimuth area. These features also demonstrated statistical significance independently (e.g., SVG peak magnitude: *p* < 0.001, with median values of 66.0 [43.4, 86.2] in the non-event group vs. 45.6 [31.4, 64.4] in the event group), supporting their clinical relevance.

Currently, patients are often referred to quaternary centers for comprehensive evaluation, including advanced diagnostics not available in primary care. The proposed model could be deployed earlier in the care pathway to help filter referrals more effectively.

Ultimately, while the integration of GEH into triage holds substantial promise, its clinical adoption will depend on overcoming several key challenges. One major hurdle is the incorporation of GEH computation into commercial ECG equipment. Although these parameters can be calculated rapidly using standard computers, embedding GEH analytics directly into clinical devices may take time ‒ especially in the absence of established technical standards for commercial implementation. In addition, successful deployment will require seamless interoperability with Electronic Health Record (EHR) systems through open standards such as HL7 and FHIR, as well as the development of clear clinical guidelines and decision-support tools to ensure safe and effective use. As emphasized by prior studies on AI-enabled ECG tools, such as Attia et al. (2019) for contractile dysfunction screening,^[^^13^ and the demonstrated reproducibility of GEH in diverse populations,^[^^12^ this field is rapidly evolving. Continued research, validation, and ethical oversight will be essential to safely and effectively integrating these innovations into routine care.

### Limitations

Implementing such approaches using real-world hospital data introduces challenges, particularly related to data imbalance. Like many clinical datasets, ours exhibited a limited number of adverse outcomes (51 of 274), which constrains statistical power and model generalizability. Although sensitivity analysis was performed, the small sample size remains a limitation. Larger, multicenter validation cohorts will be essential to confirm these findings and ensure model robustness across populations.

While the inclusion of GEH parameters improved performance compared to baseline, there is room for improvement on the overall predictive strength of the Machine Learning model. The model achieved an AUC of 67.6 % and specificity of 30.77 %, which, despite maintaining a high sensitivity of 94.12 %, may lead to over-referral in practice. This trade-off, while clinically intentional to minimize false negatives, limits the model's real-world efficiency and should be carefully optimized in subsequent studies.

Established triage tools such as the GRACE and TIMI scores provide important benchmarks for cardiovascular risk stratification. However, these metrics were not available at the primary care level in the present dataset, which represents a study limitation. Future work will aim to incorporate real-world clinical decision data to enable direct comparisons with these established tools.

While the proposed model demonstrates potential to enhance pre-referral triage, prospective validation studies integrating clinician decision data are required to confirm whether the GEH-based approach yields measurable gains in triage efficiency and patient outcomes.

Finally, there are important technical and operational challenges to clinical deployment. Real-time computation of VCG-derived GEH parameters and their integration into commercial ECG systems remain non-trivial tasks. Embedding GEH analytics into existing devices will require industry standardization, interoperability with EHR systems (HL7/FHIR), and consistent validation across manufacturers. These factors remain challenges to be addressed.

## Conclusion

The incorporation of Vectorcardiographic (VCG) features derived from standard 12-lead ECG signals may enhance the early triage and risk prediction of cardiovascular outcomes in referred populations. By leveraging automatically extractable signal-based parameters within a machine learning framework, this study presents a strategy to support decision-making in both specialized and resource-limited settings.

A key contribution of this work is the development of a robust and interpretable model that provides transparent explanations for its predictions through SHAP values. The model's output aligns well with established clinical knowledge and statistical comparisons, reinforcing the validity of its predictions. Importantly, the most influential features identified ‒ such as QRS-T angles, Spatial Ventricular Gradient (SVG), and prior cardiac events ‒ are also those shown to be significantly associated with outcomes in traditional univariate analyses. This coherence not only supports the robustness of the model but also highlights the prognostic potential of Global Electrical Heterogeneity (GEH) parameters, an area of growing interest in cardiovascular research.

Ultimately, while this approach shows promise in enhancing cardiovascular risk assessment, its clinical utility will depend on rigorous prospective validation and thoughtful alignment with current care pathways and health system infrastructures.

## Ethics approval and consent to participate

This study was conducted in accordance with the STROBE guidelines. The research protocol was reviewed and approved by the Ethics Committees of the Dante Pazzanese Institute of Cardiology and the Brazilian National Research Ethics Commission (CAAE: 76,085,317.5.000.5462). Written informed consent was obtained from all participants prior to their inclusion in the study.

## Authors’ contributions

Study concept and design: Renata Valeri de Freitas, Lucas José da Costa; Acquisition and analysis of data: Renato A. Hortegal, Mariana F. N de Marchi; Interpretation of data and Machine Learning Model: Renata Valeri de Freitas, Lucas Jos da Costa, Vinicius Ruiz Uemoto; Drafting manuscript: Lucas Jos da Costa, Vinicius Ruiz Uemoto, Renata Valeri de Freitas. All authors participated in the critical review and approval of the final draft submitted.

## Funding

This study was financed by the Fundação de Amparo à Pesquisa do Estado de São Paulo (FAPESP), process 2021/07005–4.

## Data availability

The data are available upon reasonable request from the corresponding author.

## Declaration of competing interest

The authors declare no conflicts of interest.
